# Atomization in Pulmonary Hemorrhage

**Published:** 1881-03-20

**Authors:** Willard H. Morse


### ATOMIZATION IN PULMONARY HEMORRHAGE.

BY WILLARD H. MORSE, M. D.

The question has been raised as to whether there is any real utility in the treatment of pulmonary hemorrhage by atomization. Although my experience has been quite limited, I desire to say a few words on this subject. The question admits of two antipodal answers. If the atomization be rightly conducted it is of excellent efficacy; if it be poorly conducted it is worse than useless. There are physicians fresh in their anatomy and positive in their prejudices who are not slow to assert that the treatment is without the least advantage, they arguing that foreign substances cannot pass the chink of the glottis without causing deleterious effects. Nevertheless, over and above a possibility of controversy the fact is undeniable that although medicated spray is of the nature of a foreign substance, it can and does pass the glottis, and instead of having any injurious effect, is beyond the shadow of a doubt of sterling utility in bringing about the end sought. Despite the contrary assertions physiological experiments have conclusively established the fact that minute quantities of the spray can enter the trachea. But I cannot believe that the efficiency of atomization depends entirely on the amount of spray that enters the glottis, but is due in no small part to the evidence of sympathy.

There are three medical agents that claim our attention as applicable in pulmonary hemorrhage—perchloride and subsulphate of iron, tannin, and alum. There may be other substances equally capable of atomization, and equally as applicable in controlling the hemorrhage, but they are untried, or if tried have been found wanting in the balance. The iron, tannin, and alum are alone proved to the truth. The most efficient of the three is the one first named. Tannin takes a second place, and scarcely less reliable is the alum solution. Much of the relative efficacy of the three depends upon the strength in which they are used. Of the ferric perchloride I consider a half-to two-grain solution amply sufficient to meet the wants of any case. The tannin may be given from two to twenty-five grains, and the alum from ten to twenty. Although these are not the dispensatory doses, I think them better; but of course the dosage is to be governed by the case. Another essential, and one which I think has been overlooked and underestimated, has reference to the water of the solution. It will be found that if the menstruum be at a heat of 70° to 80° F. it will be less apt to irritate the lungs, and have more perfect "sympathetic" action. It is also too frequently the case in the emergency of the disease to use any water that comes to the hand, provided it is not at boiling heat. Under such circumstances failure is not rare, whereas a uniform heat of 70° or 80° F., having no condition of irritation in temperature, is more conclusive to utility.

Another requisite to success in treatment relates to the amount of a given solution required to have a styptic effect. Considering that only a very minute quantity of the solution enters upon the affected broncho-pulmonary tract, it seems idle to inquire in this regard. Yet admitting that the primary effect of the action of the spray is extraneous by choice, it is well to fix some relative administration. The ferric solution should not be given without some definite intermission—five or ten minutes being necessary after a spraying of a half dram of the solution—that in that time the effect of the medicine may be noted. The tannin solution rarely requires to be used more than a minute at a time, six or eight such sprayings being amply sufficient in favorable cases.

Another requisite to success consists in the instrument used. I never employ the steam atomizer, as it is rarely if ever ready for use in such an emergency as pulmonary hemorrhage offers. Of the hand-ball atomizers I prefer the Delano instrument, and especialy the "No. 358," which is made with an extra long tube, and which is *par excellence* the best atomizer for atomization in diphtheria, catarrh, bronchitis, or any "throat disease" requiring nebulization, as well as in the pulmonary hemorrhage.

Mode of application of the spray is another essential. It is advisable to introduce the tube as far as the middle of the tongue and give it the proper downward direction, so that the force of the volume of spray a distance of an inch from the nozzle of the delivery-tube shall strike in front and across the epiglottis.

Another correlative rule of application is promptness of action. Delay is more than dangerous. Early attention determins all. There may be cases where any amount of atomization would be useless, but still local measures *may* do good, and it is always best to employ the atomization. Make no prejudicial choice of the agent to use, giving iron or tannin or alum, as it may be at hand, always remembering, however, that ferric solutions are of most merit.

If the precautions of which I have spoken be observed, atomization will be attended with success; but we should always bear in mind that it is like faith, utterly "dead" without the "works" of general therapeutical measures. A favorable formula of mine is—

Fluid. ext. ergot (Squibb's)........................................ ℥ iij :

Tinct. opii deodor........................................................ }

Fluid ext. ipecac.......................................................... } aa ℥ ss.

M. Sig. Teaspoonful every half hour in connection with the use of atomization.—*Louisville Medical News.*

---

The Italian government is about to impose heavy duties on cotton-seed oil, to prevent its being used for the adulteration of olive oil, a practice which is fast ruining the Italian oil trade.

